# Tuberculosis case finding using population-based disease surveillance platforms in urban and rural Kenya

**DOI:** 10.1186/s12879-018-3172-z

**Published:** 2018-06-07

**Authors:** Godfrey Bigogo, Kevin Cain, Diana Nyole, Geoffrey Masyongo, Joshua Auko Auko, Newton Wamola, Albert Okumu, Janet Agaya, Joel Montgomery, Martien Borgdorff, Deron Burton

**Affiliations:** 1 Centre for Global Health Research, Kenya Medical Research Institute, P.O Box 1578 -, Kisumu, 40100 Kenya; 2 U.S. Centers for Disease Control and Prevention, Atlanta, USA; 3 Academic Medical Centre, University of Amsterdam, Amsterdam, Netherlands

**Keywords:** Tuberculosis, Case-finding, Kenya

## Abstract

**Background:**

Tuberculosis (TB) case finding is an important component of TB control because it can reduce transmission of *Mycobacterium tuberculosis* (MTB) through prompt detection and treatment of infectious patients.

**Methods:**

Using population-based infectious disease surveillance (PBIDS) platforms with links to health facilities in Kenya we implemented intensified TB case finding in the community and at the health facilities, as an adjunct to routine passive case finding conducted by the national TB program. From 2011 to 2014, PBIDS participants ≥15 years were screened either at home or health facilities for possible TB symptoms which included cough, fever, night sweats or weight loss in the preceding 2 weeks. At home, participants with possible TB symptoms had expectorated sputum collected. At the clinic, HIV-infected participants with possible TB symptoms were invited to produce sputum. Those without HIV but with symptoms lasting 7 days including the visit day had chest radiographs performed, and had sputum collected if the radiographs were abnormal. Sputum samples were tested for the presence of MTB using the Xpert MTB/RIF assay. TB detection rates were calculated per 100,000 persons screened.

**Results:**

Of 11,191 participants aged ≥15 years screened at home at both sites, 2695 (23.9%) reported possible TB symptoms, of whom 2258 (83.8%) produced sputum specimens. MTB was detected in 32 (1.4%) of the specimens resulting in a detection rate of 286/100,000 persons screened. At the health facilities, a total of 11,762 person were screened, 7500 (63.8%) had possible TB symptoms of whom 1282 (17.1%) produced sputum samples. MTB was detected in 69 (5.4%) of the samples, resulting in an overall detection rate of 587/100,000 persons screened. The TB detection rate was higher in persons with HIV compared to those without at both home (HIV-infected - 769/100,000, HIV-uninfected 141/100,000, rate ratio (RR) – 5.45, 95% CI 3.25–22.37), and health facilities (HIV-infected 3399/100,000, HIV-uninfected 294/100,000, RR 11.56, 95% CI 6.18–18.44).

**Conclusion:**

Facility-based intensified TB case finding detected more TB cases per the number of specimens tested and the number of persons screened, including those with HIV, than home-based TB screening and should be further evaluated to determine its potential programmatic impact.

## Background

A central aim of tuberculosis (TB) control programs is to quickly identify new TB cases and ensure that these cases successfully complete treatment [1–3]. Prompt treatment of persons with TB disease halts their infectiousness and reduces transmission of TB, resulting in a decline in TB incidence in the community [4]. Case detection is an important component in TB programs, but it can be influenced by several factors including health care access, care seeking practices and diagnostic capabilities [5–7]. TB programs in many countries rely primarily on passive TB case finding where patients self-refer to clinics and are screened for TB based on the attending clinician’s judgment. However, in many parts of the developing world with poor access to health facilities, passive case finding alone may not achieve the global target of 70% case detection rate [1, 8, 9]. In sub-Saharan Africa, TB diagnosis and treatment have not been prompt with delays ranging from 50 to 180 days reported [5, 6, 10–12].

Early detection and treatment are important in reducing transmission of the tuberculosis bacilli [13–15]. Intensified case finding among household members of infectious TB cases is an effective approach to curb TB spread [16, 17]. However in areas with high rates of TB and HIV, important sources of infection may be contacts outside the household, thus a broader, community-based approach combined with enhanced screening at health facilities may be needed to improve TB case finding in such settings [9, 14, 15, 18–23]. We examined the yield of intensified TB case finding at community level and in health facilities among persons ≥15 years of age as an adjunct to the passive facility based case detection method in areas with high TB and HIV prevalence in urban and rural Kenya.

## Methods

### Study sites

**Fig. 1:**
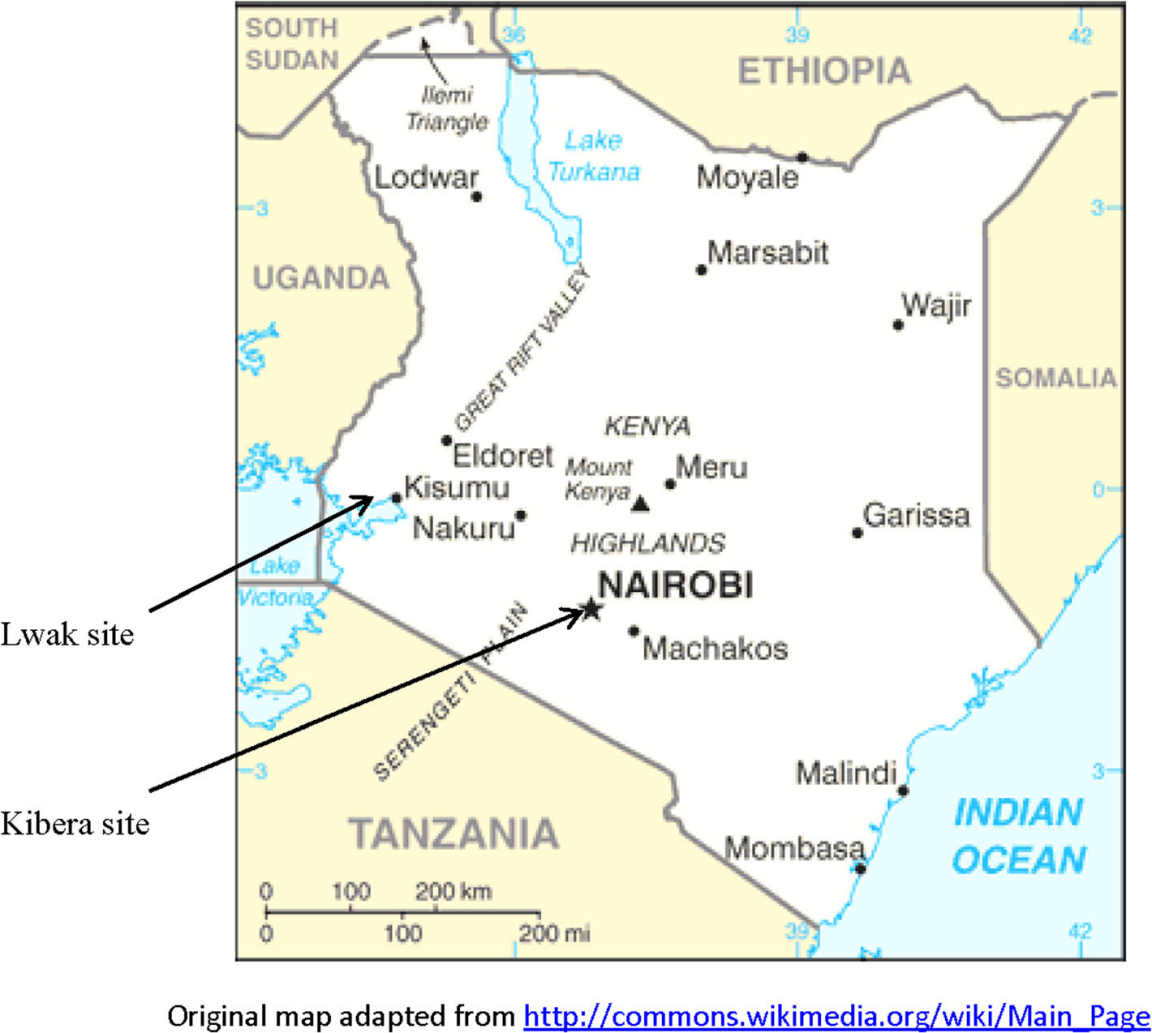
Map of Kenya with location of study sites

The Kenya Medical Research Institute (KEMRI), in collaboration with the Centers for Disease Control and Prevention (CDC) have conducted population-based infectious disease surveillance (PBIDS) in two sites in Kenya since 2005; in Kibera, an urban slum in Nairobi, and in Lwak in rural western Kenya (Fig. 1) [24, 25]. The two study sites have referral facilities – Lwak hospital in the rural site and Tabitha clinic in the urban site – where participants can access free care for all potentially infectious disease syndromes, and can access TB clinics that operate 5 days a week (Monday – Friday). Besides the TB clinics at the referral facilities, there were several other TB clinics run by the Ministry of Health within the PBIDS areas. Some PBIDS participants visited these clinics for care.

### Kibera site

The surveillance area located in Kibera is < 0.5km^2^, and has a population between 25,000 and 29,000 since PBIDS began; population density is 77,000 people per km^2^. Nearly all (98%) residents in the area under surveillance are enrolled in PBIDS [24]. Residents live in houses built of mud or brick walls with tin roofs. Open sewers flow along footpaths and water is mostly obtained from vendors. Most individuals living in the slum work as casual laborers or engage in small businesses. HIV prevalence in the area in the years 2009–2010 was 15% among individuals aged ≥18 years [26]. Prior to the implementation of intensified TB case finding, baseline data abstracted from TB registers from health facilities in the study area indicated an annual TB notification rate for persons ≥15 years of 567 cases per 100,000 persons between 2009 and 2011 (unpublished PBIDS data). Other than Tabitha clinic, there are two additional clinics within the study area that offered TB screening (through sputum microscopy) of patients based on clinicians’ assessments.

### Lwak site

The Lwak site is situated in Siaya County in rural western Kenya. The study site and the socio-economic characteristics of the population in this area have been described previously [25, 27, 28]. Briefly, the PBIDS catchment population approximated at 24,000–26,000 is drawn from individuals living in 33 villages within a 5 km radius of Lwak hospital, a private hospital that serves as the project’s referral facility. The population density is 320 people per km^2^. Besides Lwak hospital, there are three government-run health facilities which conduct passive TB case finding in the area. A TB prevalence study done in the area in the years 2006–2008 found a prevalence of 600 cases per 100,000 persons [29]. Baseline PBIDS data estimated a TB notification rate of 706 cases per 100,000 persons for the years 2009–2011. HIV prevalence in the years 2008–2009 was estimated at 17% among adults [26].

## Data collection and analysis

### Household TB screening and specimen collection

As part of ongoing disease surveillance since 2005, participants in the PBIDS project were visited weekly or bi-weekly at home by field workers who used standardized questionnaires to gather information about any illnesses since the last home visit [25, 30–32]. At the onset, the study focused on characterization of four syndromes, which were pneumonia, diarrhea febrile illness and jaundice. Data on illnesses and care seeking for reported illnesses were recorded in programmed personal digital assistants (PDAs). Specific symptoms of illness asked for included cough, fever, difficulty in breathing, diarrhea, and yellow eyes. Abbreviated physical exams including axillary temperature measurements were carried out on ill persons present during the visit. Characterization of TB was not part of the original scope of the PBIDS surveillance project, and positive responses to the original routine symptom questions were not used to make referrals for TB testing. Rather, positive responses to the original symptom questions were used to make referrals for further evaluation of the four syndromes mentioned above.

From August to December 2011 for the Lwak site and from January to April 2012 for the Kibera site, active home screening for TB was conducted in which additional symptoms, including presence of weight loss and night sweats, were collected. Participants aged ≥15 years who reported any cough, fever or night sweats in the previous 2 weeks, or weight loss over the month prior to the interview date were considered to have possible TB. They were consented and requested to produce sputum specimens in private inside their houses, or in the open outside of their houses. They were taught how to make a “huff cough” to help produce sputum. Field workers stored the sputum samples in boxes with ice packs which were maintained at + 2 – + 8 °C. Sample collectors picked up the samples from the field workers and ferried them to a central location at Lwak hospital or Tabitha clinic, where the samples were registered in logs, batched and stored in refrigerators at + 2 − + 8 °C awaiting shipment to KEMRI laboratories in Kisumu or Nairobi.

### Facility-based TB screening and specimen collection

**Fig. 2:**
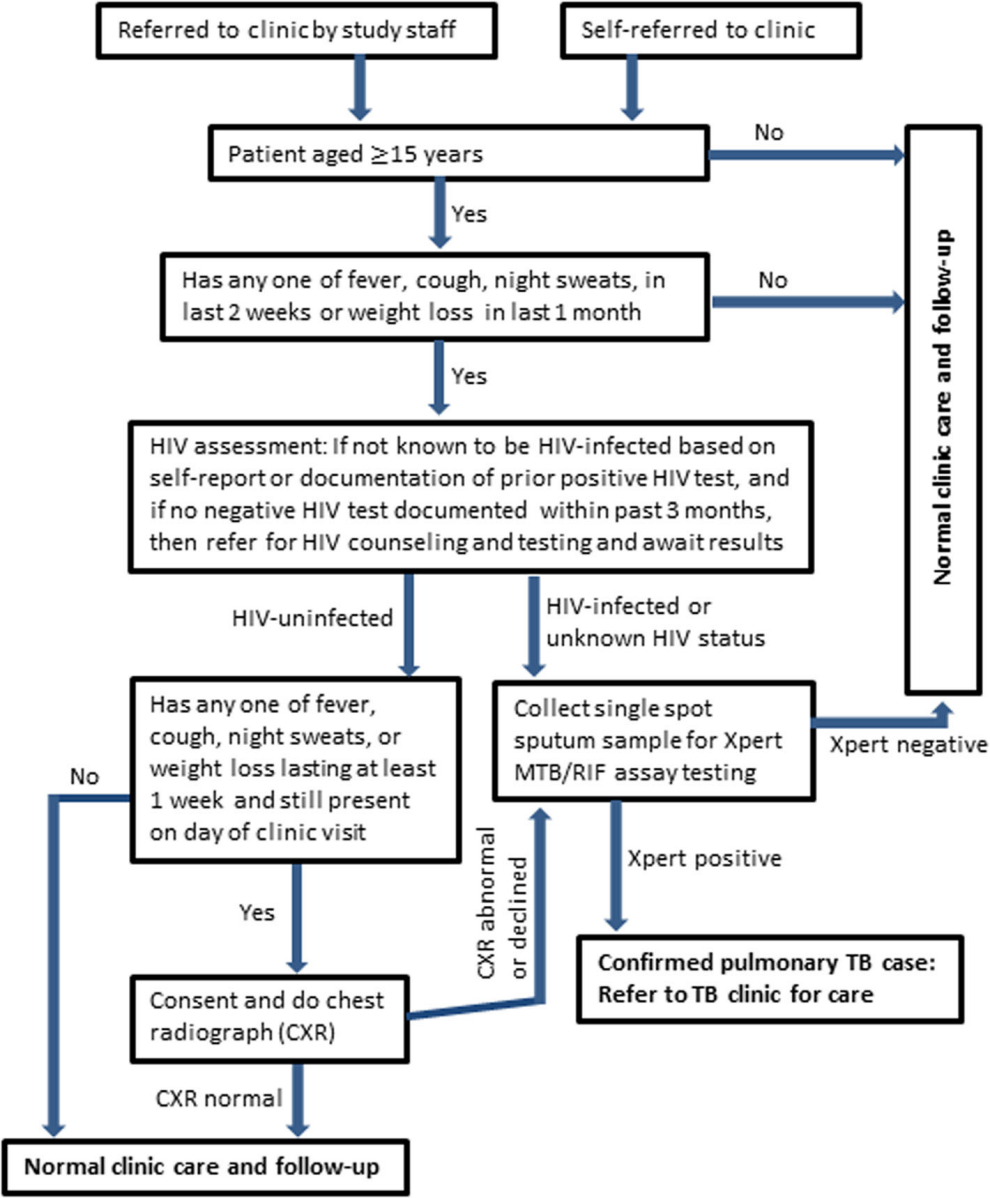
Facility based TB screening flowchart used at the PBIDS health facilities in Lwak and Kibera

PBIDS participants aged ≥15 years old who presented to Tabitha clinic (from April 2012 to March 2014) or Lwak hospital (from May 2012 to April 2014) were asked if they had any of the following four possible TB symptoms: cough, fever, night sweats or weight loss in the last 1 month. Questions about the latter two symptoms were added as part of the TB case-finding intervention. Any participant reporting any of these symptoms was evaluated further according to a set algorithm (Fig. 2). In brief, HIV-infected participants with at least one of these symptoms were invited to produce sputum. Participants without HIV but with possible TB symptoms lasting at least 7 days including the day of the sick visit had a chest radiograph performed and were asked for sputum if the radiograph was abnormal. Sputum samples were logged and stored in refrigerators at + 2 − + 8 °C until shipment to the study laboratory.

### Laboratory testing and follow-up

Sputum samples were delivered daily from the study clinics to the KEMRI laboratories in Kisumu or Nairobi. Testing of the samples was done by Xpert MTB/RIF assay, which uses molecular techniques to detect the presence of *Mycobacterium tuberculosis* (MTB) and determine whether the bacterium is resistant to rifampicin [33, 34]. Test results were available after 2 h. A confirmed pulmonary TB case was defined by a single positive Xpert result. Smear microscopy was performed on specimens from confirmed pulmonary TB cases in accordance with national TB program policy in Kenya [35].

All febrile patients had malaria tests and a subset had blood cultures done as described elsewhere [25]. Those with severe acute respiratory illness had blood cultures done [36].

Laboratory results were linked to participants’ clinical data collected at home or at the study facilities. All TB results (positive and negative) were disseminated back to the participants by field workers within 2 weeks. Participants with confirmed pulmonary TB were invited to come to the study facilities and were referred to the onsite TB clinics, or were asked to visit a TB clinic of their choice for further evaluation and care according to Kenya’s national TB program policy [35]. All positive TB test results were shared with the Kenyan Ministry of Health, Division of TB and Leprosy. Participants with negative TB test results but who still experienced possible TB symptoms 1 month after initial TB testing were invited to be retested.

### HIV status data

Large-scale home based HIV counseling and testing (HBCT) was performed in the years 2008–2009 when all participants ≥13 years in the PBIDS areas were offered HIV testing as described previously [26, 37]. Seventy-eight percent of eligible adults ≥18 years and 87% of youth aged 13–17 years agreed to be tested during that period [26]. In subsequent years, HIV testing targeted adult new PBIDS participants and previously HIV-uninfected residents who requested to be re-tested. For those participants not retested after the home-based HIV counseling and testing campaign, we assumed that their HIV status at the time of home-based testing was the same throughout the study period. This assumption is supported by the low estimated annual HIV transmission in the study areas (< 1% based on KEMRI unpublished data).

HIV counseling and testing were offered routinely at the study referral facilities for all participants aged ≥15 years. For this project, previous HIV positive results were accepted. Participants with previous HIV negative results > 3 months old or those with an unknown HIV status were invited to be tested for HIV infection during the period of intensified TB case finding.

### Data analysis

We describe demographic and clinical characteristics of PBIDS participants with confirmed pulmonary TB at the two study sites. TB detection rates were calculated as the number of cases per 100,000 persons screened, disaggregated by age group, gender, and HIV status. We used Poisson regression to calculate rate ratios and compare TB detection rates by age, gender and HIV status among persons screened at home and at study health facilities. We controlled for clustering of cases at household level using general estimating equations. For this analysis we assumed an individual’s HIV status at home screening was the same as that established at HBCT unless there were different results from more recent tests. Analyses were done using STATA 13.1 Software (StataCorp, Texas USA).

### Ethical consideration

The protocol was reviewed and approved by the Kenya Medical research Institute (KEMRI) Ethics Review Committee (No. 1899) and the Centres for Disease Control and Prevention (CDC) Institutional Review Board (No. 4566). All participants provided consent to participate in the study. Participants aged 15–17 years provided assent after parental consent was obtained.

## Results

### Screening for possible TB symptoms and results of TB sputum testing

At home, 6575/13,740 (47.9%) and 4616/12,864 (35.9%) of PBIDS participants aged ≥15 years in Lwak and Kibera, respectively, were available for TB symptom screening. Of the total 11,191 PBIDS participants screened at home, 2695 (24.1%) reported possible TB symptoms of whom 2258 (83.8%) had sputum successfully collected. Of the participants with sputum available for testing, 32 (1.4%) participants (28 at initial round and 4 at retesting 1 month later) were confirmed to have pulmonary TB. TB positivity of sputum specimens collected at home was similar across the two sites; 1.2% (18/1546) in Lwak (Table 1), and 2.0% (14/712) in Kibera (Fisher’s exact *p*-value = 0.178).

**Table 1:** Detection of pulmonary TB among participants screened in population-based surveillance sites in Lwak and Kibera in Kenya, 2011–2014

	AT HOME							AT CLINIC						
	Resident population N	Pop. Screened n (% of resident pop)	Possible TB cases^a^ n (% of pop. screened)	Sputum obtained n (% of possible TB cases)	Confirmed TB cases n (% of those with sputum obtained)	TB detection rate per 100,000 persons screened (95% CI)	Prevalence ratio (95% CI)	Resident population N	Pop. Screened n (% of resident pop)	Possible TB cases^a^ n (% of population screened)	Sputum obtained n (% of possible TB cases)	Confirmed TB cases n (% of those with sputum obtained)	TB detection rate per 100,000 persons screened (95% CI)	Prevalence ratio (95% CI)
a) Lwak														
Overall	13,740	6575 (47.9)	1804 (27.4)	1546 (85.7)	18 (1.2)	274 (162–432)		13,800	5765 (41.8)	4242 (73.6)	607 (14.3)	28 (4.6)	486 (323–701)	
Age (yrs)														
15–34	7816	2613 (33.4)	521 (19.9)	422 (81.0)	4 (0.9)	153 (42–392)	Ref	7565	3315 (43.8)	2399 (72.4)	227 (9.5)	15 (6.6)	452 (254–745)	Ref
≥ 35	5924	3962 (66.9)	1283 (32.4)	1124 (87.6)	14 (1.2)	353 (193–592)	2.31 (0.76–7.00)	6235	2450 (39.3)	1843 (75.2)	380 (20.6)	13 (3.4)	531 (283–906)	1.17 (0.56–2.46)
Gender														
Female	7617	4716 (61.9)	1289 (27.3)	1107 (85.9)	11 (1.0)	233 (117–417)	Ref	7588	3811 (50.2)	2853 (74.9)	401 (14.1)	12 (3.0)	315 (163–549)	Ref
Male	6123	1859 (30.4)	515 (27.7)	439 (85.2)	7 (1.6)	377 (152–774)	1.61 (0.63–4.16)	6212	1954 (31.4)	1389 (71.1)	206 (14.8)	16 (7.8)	819 (469–1326)	2.60 (1.23–5.49)
HIV														
HIV-Neg	6519	3332 (51.1)	974 (29.2)	850 (87.3)	4 (0.5)	120 (33–307)	Ref	8857	3634 (41.0)	3099 (85.3)	153 (4.9)	3 (2.0)	83 (17–241)	Ref
HIV-pos	1143	761 (66.6)	235 (30.9)	211 (89.8)	6 (2.8)	788 (290–1708)	6.57 (1.86–23.22)	1716	891 (51.9)	808 (90.7)	437 (54.1)	24 (5.5)	2694 (1733–3982)	32.63 (9.85–108.11)
Unknown	6078	2482 (40.8)	595 (24.0)	485 (81.5)	8 (1.6)	322 (139–634)	2.68 (0.81–8.91)	3227	1240 (38.2)	335 (27.0)	17 (5.1)	1 (5.9)	81 (2–449)	0.98 (0.10–9.38)
	AT HOME							AT CLINIC						
	Resident population N	Pop. Screened n (% of resident pop.)	Possible TB cases^b^ n (% of pop. screened)	Sputum obtained n (% of possible TB cases)	Confirmed TB cases n (% of those with sputum obtained)	TB detection rate per 100,000 persons screened (95% CI)	Prevalence ratio (95% CI)	Resident population N	Pop. Screened n (% of resident pop.)	Possible TB cases^b^ n (% of population screened)	Sputum obtained n (% of possible TB cases)	Confirmed TB cases n (% of those with sputum obtained)	TB detection rate per 100,000 persons screened (95% CI)	Prevalence ratio (95% CI)
b) Kibera														
Overall	12,864	4616 (35.9)	891 (19.3)	712 (79.9)	14 (2.0)	303 (164–508)		11,448	5997 (52.4)	3258 (54.3)	675 (20.7)	41 (6.1)	684 (491–926)	
Age (yrs)														
15–34	9115	3218 (35.3)	529 (16.4)	410 (77.5)	9 (2.2)	280 (128–530)	Ref	7503	4229 (56.4)	2303 (54.5)	414 (18.0)	24 (5.8)	568 (364–843)	Ref
≥ 35	3749	1398 (37.3)	362 (25.9)	302 (83.4)	5 (1.7)	358 (116–833)	1.28 (0.43–3.81)	3945	1768 (44.8)	955 (54.0)	261 (27.3)	17 (6.5)	962 (561–1535)	1.69 (0.91–3.15)
Gender														
Female	6947	3220 (46.4)	614 (19.1)	491 (80.0)	9 (1.8)	280 (128–530)	Ref	5693	3743 (65.7)	2106 (56.3)	387 (18.4)	18 (4.7)	481 (285–759)	Ref
Male	5917	1396 (23.6)	277 (19.8)	221 (79.8)	5 (2.3)	358 (116–834)	1.28 (0.43–3.82)	5755	2254 (38.4)	1152 (51.1)	288 (25.0)	23 (8.0)	1020 (648–1527)	2.12 1.15–3.92)
HIV														
HIV-Neg	3084	927 (30.1)	247 (26.6)	194 (78.5)	3 (1.5)	324 (67–943)	Ref	4979	2830 (56.8)	1835 (64.8)	213 (11.6)	16 (7.5)	565 (324–917)	Ref
HIV-pos	371	149 (40.2)	37 (24.8)	25 (67.6)	6 (24.0)	4027 (1768–7793)	12.44 (3.15–49.22)	662	374 (56.5)	286 (76.5)	158 (55.2)	19 (12.0)	5080 (3086–7820)	8.99 (4.66–17.32)
Unknown	9409	3540 (37.6)	607 (17.1)	493 (81.2)	5 (1.0)	141 (46–329)	0.44 (0.10–1.82)	5799	2793 (48.2)	1137 (40.7)	304 (26.7)	6 (2.0)	215 (79–467)	0.38 (0.15–0.97)

^a^Possible TB cases = Anyone meeting any of the following: any cough, fever, or night sweats in last 2 weeks, or weight loss in last one month. Data at home collected between October – December 2011. Clinic data collected between May 2012 – April 2014. For clinic, resident population is at 30th April 2014. CI=Confidence Interval

^b^Possible TB cases = Anyone meeting any of the following: any cough, fever, night sweats in last 2 weeks or weight loss in last one month. Data at home collected between January – March 2012. Clinic data collected between April 2012 – March 2014. For clinic, resident population is at 31st March 2014. CI=Confidence Interval

At the study health facilities 5765/13,800 (41.8%) and 5997/11,448 (52.4%) of the resident populations were screened in Lwak and Kibera, respectively. Of the total 11,762 participants screened at the two health facilities, 7500 (63.8%) were found with possible TB symptoms, of whom 1282 (17.1%) had sputum collected. TB was detected in 69 (5.4%) of the sputum samples obtained at the health facilities. TB positivity of sputum specimens was 4.6% (28/607) at Lwak hospital (Table 1), and 6.1% (41/675) at Tabitha clinic in Kibera (Fisher’s exact *p*-value = 0.266).

### Demographic and clinical characteristics of participants with confirmed pulmonary TB

Of the 101 participants confirmed to have pulmonary TB across both study sites (32 from home-based screening, 69 from facility-based screening), 51 (50%) were males and 55 (54%) were HIV-infected (Table 2). The mean age of participants with confirmed TB was 37 years (range 19–77 years). Cough was the most common symptom reported in 75 (74%) of the confirmed pulmonary TB cases. Overall, 49.5% (50/101) of participants with confirmed pulmonary TB had positive sputum smear microscopy, ranging from 35.7% (10/28) among those screened for TB at Lwak hospital to 57.1% (8/14) among those screened at home in Kibera. Among participants with confirmed pulmonary TB at the health facilities who had blood cultures (13 total) or blood smears for malaria (17 total) done, two patients in each category were co-infected (15% positive blood cultures; 12% positive malaria smear). All participants with confirmed pulmonary TB (101/101) were referred to TB clinics for care and the Ministry of Health was notified of their TB test results.

**Table 2:** Characteristics of participants ≥15 years with confirmed pulmonary TB in Lwak and Kibera study sites in Kenya, 2011–2014

Lwak			Kibera		
	From home screening^a^	From facility-based screening^b^	From home screening^a^	From facility-based screening^b^	Total
	*N* = 18	*N* = 28	*N* = 14	*N* = 41	*N* = 101
	n(%)	n(%)	n(%)	n(%)	n(%)
Mean age	51	41	34	33	37
(Min – max)	(23–77)	(21–56)	(24–54)	(19–59)	19–77
Gender					
Male	7 (38.9)	16 (57.1)	5 (35.7)	23 (56.1)	51 (50.5)
HIV status					
Positive	6 (33.3)	24 (85.7)	6 (42.9)	19 (46.3)	55 (54.5)
Negative	4 (22.2)	3 (10.7)	3 (21.4)	16 (39.0)	26 (25.7)
Unknown	8 (44.4)	1 (3.6)	5 (35.7)	6 (14.6)	20 (19.8)
Socioeconomic status^c^					
Low	9 (50.0)	5 (17.9)	–	–	14/46 (30.4)
Middle	4 (22.2)	16 (57.1)	–	–	20/46 (43.5)
High	5 (27.8)	6 (21.4)	–	–	11/46 (23.9)
Not available	0 (0)	1 (3.6)	–	–	1/46 (2.2)
Previous history of TB^d^	4 (22.2)	–	3 (21.4)	–	7/32 (21.9)
Symptoms reported					
Cough	16 (88.9)	20 (71.4)	9 (64.3)	30 (73.2)	75 (74.3)
Fever	3 (16.7)	15 (53.6)	6 (42.9)	6 (14.6)	30 (29.7)
Night sweats	6 (33.3)	8 (28.6)	5 (35.7)	5 (12.2)	24 (23.8)
Weight loss	4 (22.2)	7 (25.0)	5 (35.7)	5 (12.2)	21 (20.8)
Duration of symptoms^e^					
≤ 2 weeks	14 (77.8)	–	8 (57.1)	–	22 (21.8)
> 2 weeks	4 (22.2)	–	6 (42.9)	–	10 (9.9)
Coinfections^f^					
Positive blood culture	–	2/13 (15.4)	–	–	2/13 (15.4)
Positive malaria smear	–	1/16 (6.3)	–	1/1 (100)	2/17 (11.8)
Smear positive TB	9 (50)	10 (35.7)	8 (57.1)	23 (56.1)	50 (49.5)
MoH Notified	18 (100)	28 (100)	14 (100)	41 (100)	101 (100)
Referred to TB clinic	18 (100)	28 (100)	14 (100)	41 (100)	101 (100)

^a^TB screening at home occurred in October – December 2011 for Lwak, and January – March 2012 for Kibera, and the column contains only individuals screened in-person

^b^Facility-based TB screened occurred between May 2012 – April 2014 for Lwak, and April 2012 – March 2014 for Kibera. Individuals were referred from home or could self-refer to the health facilities

^c^Socioeconomic status classes computed based on asset ownership were available for Lwak site only

^d^^,^^e^Data available from home only

^f^Data available for health facilities only. Denominators are the number of persons who had either a blood culture or a malaria test done among those who had a positive TB result

### Detection rate of confirmed pulmonary TB among screened participants according to study site, screening location, and individual characteristics

The detection rate of confirmed pulmonary TB among persons screened in person at home was 274/100,000 persons (95% CI 162–432/100,000 persons) in Lwak and 303/100,000 persons (95% CI 164–508/100,000 persons) in Kibera (Table 1). At the health facilities, the TB detection rate in Lwak hospital was 486/100,000 persons (95% CI 323–701/100,000 persons), and in Tabitha clinic was 684/100,000 persons (95% CI 491–926/100,000 persons) (Table 1). Overall, across both study sites, the TB detection rate was significantly higher for screening performed at the referral facilities compared to homes (referral facility TB detection rate: 587/100,000 persons, 95% CI 457–742/100,000 persons; home-based TB detection rate: 286/100,000 persons, 95% CI 196–403/100,000 persons; rate ratio (RR) 2.05, 95% CI 1.35–3.12).

At the health facilities, TB detection rates were approximately 2-fold higher in men than women (RR 2.60, 95% CI 1.23–5.49 in Lwak hospital; RR 2.12, 95% CI 1.15–3.92 in Tabitha clinic) (Table 1). The TB detection rate did not differ by gender for home-based TB screening at either site. For both facility-based and home-based screening, at both sites, there was a trend towards higher TB detection rates among persons aged ≥35 years compared to those aged 15–34 years, though these trends were not statistically significant (Table 1).

In home-based screening at Lwak, the TB detection rate among persons infected with HIV was 6-fold higher compared to those without HIV; 788/100,000 (95% CI 290–1708/100,000) among HIV-infected persons compared to 120/100,000 (95% CI 33–307) among HIV-uninfected persons, RR 6.57 (95% CI 1.86–23.22). The detection rate of 4027/100,000 (95% CI 1768–7793/100,000) among HIV-infected persons screened at home in Kibera was 12-fold higher compared to the rate of 324/100,000 (95% CI 67–943) among HIV-uninfected persons; RR 12.44, (95% CI, 3.15–49.22) (Table 1).

In facility-based TB screening across study sites, TB detection rates among persons infected with HIV were 9- and 33-fold higher compared to those without HIV in Kibera and Lwak respectively (Table 1). In Lwak the rate was 2694/100,000 (95% CI 1733–3982/100,000) persons among HIV-infected persons compared to 83/100,000 (95% CI 17–241/100,000) persons, among HIV-uninfected; RR 32.63 (95% CI 9.85–108.11). In Kibera the detection rate was 5080/100,000 persons (95% CI 3086–7820/100,000 persons) among HIV-infected persons compared to 565/100,000 (95% CI 324–917/100,000), among HIV-uninfected; RR 8.97 (95% CI, 4.66–17.32).

## Discussion

We implemented community- and health facility-based active TB case-finding using existing population-based infectious disease surveillance platforms in rural and urban sites in Kenya. In both sites we observed greater TB detection rates among participants screened for TB at health facilities compared to those screened at home. Our study was unique in applying similar TB case finding strategies in well-established rural and urban PBIDS sites with distinct demographic and epidemiologic profiles using electronic data collection systems, as adjuncts to the Ministry of Health’s passive TB case detection methods. The detailed characterization of study participants within the PBIDS platforms enabled us to calculate screening coverage and TB detection rates stratified by various demographic and clinical characteristics, and the close proximity of referral health facilities and KEMRI laboratories facilitated rapid TB diagnosis using the Xpert MTB/RIF assay, which at the time of the study was not widely available in many areas in Kenya.

At both study sites, overall TB detection rates and the proportion of sputum specimens that were positive for TB were higher in participants screened at health facilities compared to those screened at home, suggesting that intensified TB case finding at health facilities may be a more efficient approach than home-based TB screening. Nevertheless, in settings with low health care seeking, home-based or other forms of community-based TB active case-finding might be important complementary approaches to facility-based TB case-finding, particularly for high-risk populations such as persons living with HIV, who had high TB detection rates at home in both of our study sites (788 and 4027 per 100,000 persons screened in the rural and urban site, respectively).

Community-based case finding strategies also have the potential to identify persons with TB early in the course of their disease and link them to treatment [9, 22], which may produce better treatment outcomes and reduce community transmission. In both our rural and urban study sites, the majority of participants diagnosed with TB through home-based intensified case finding reported symptoms that began ≤2 weeks prior to screening. Although it is not known how long these participants identified at home with confirmed pulmonary TB would have remained undetected through routine passive, facility-based TB case finding, the observation that 50–57% of TB cases identified at home were smear positive suggests that there is a strong possibility that these participants might have contributed to additional TB transmission in the community before seeking care. Given that delays in diagnosis have been identified as one of the obstacles in the control of TB [6, 11], intensified home-based TB case finding may be an important tool in TB control in appropriate settings and should be further evaluated to determine its long-term impact on TB incidence through its utility in early diagnosis of TB.

We found very high TB detection rates among persons living with HIV, both at home (1319, 95% CI 683–2292 per 100,000 persons) and at the referral health facilities (3399, 95% CI 2471–4552 per 100,000 persons). In sub-Saharan Africa, there is a virulent synergy between HIV and TB [16, 17, 22]. Of the 9.6 million incident cases of TB in 2014, an estimated 1.15 million (12%) were HIV positive; 74% of these HIV-positive cases were in the African region [3]. Our rural and urban PBIDS sites have a high prevalence of HIV estimated at 15% for persons ≥13 years [26]. In similar settings, it may be particularly beneficial to implement multifaceted intensified TB case finding among persons living with HIV, including both community-based and health facility-based strategies. Notably, uptake of sputum collection was significantly increased among persons living with HIV screened at home (87%) versus screened at the health facilities (55%). It is unclear whether this difference reflected operational issues around sputum collection at the health facilities, or increased participant acceptance of sputum collection in more private home settings, or other factors, but it is possible that TB detection rates from intensified case finding at the health facilities would have been substantially increased if a greater proportion of eligible HIV-infected participants had provided sputum samples. Effective targeting of intensified TB case finding to persons living with HIV requires robust HIV counseling and testing services with high community uptake. Strategies such as home based counseling and testing for HIV have been deployed in several African countries and have proved successful in identifying HIV infected persons [38, 39]. TB case finding efforts at community level may be aligned with HBCT efforts to increase success in identifying persons co-infected with TB and HIV.

Among participants without HIV, the TB detection rate in the urban site was greater than in the rural site for both home-based and facility-based intensified case finding. Several studies have documented high TB burden in slum settings. In a study conducted in a densely populated informal settlement in Dhaka, Bangladesh, the prevalence of TB was four-fold higher than that recorded in other urban areas with less dense populations [40]. Similarly, in an informal settlement in Site-M in Cape Town in South Africa, the TB incidence was significantly higher in densely populated areas than less dense regions [41]. Kibera in Nairobi, where our urban PBIDS site is located, is one of the largest slums in Africa and is 240-times more densely populated than our rural PBIDS site in western Kenya. It is likely that crowded living conditions increases TB transmission in the urban site.

Our home-based intensified TB case finding strategy was designed to be an adjunct to the existing passive, facility-based case finding system used by the national TB program in Kenya. While the home-based case-finding strategy was successful in quickly identifying several TB cases over a short span of time at both rural and urban sites and linking them to treatment, the overall yield from this strategy was low relative to what might have been expected based on earlier estimates of TB prevalence in the study sites [29]. The reasons for the relatively low numbers of TB cases identified at home are not clear, but it is likely that we missed some participants who were symptomatic and eligible for TB testing but were absent from home at the time of the TB screening visits. For instance, most of the participants screened at both home and the health facilities were women, but the TB detection rate was consistently increased among men, particularly at the health facilities where the TB detection rate was 2-fold greater compared to women. In some settings, home-based intensified TB case finding strategies may need to be supplemented with central community-level TB screening methods (such as mobile testing units) with extended hours to increase uptake among potentially high-risk sub-populations that are less likely to be found at home during the day [29].

Another potential limitation of our home-based screening method is that possible TB symptoms that were not present at the time of the home visit may have been underreported because of limited recall, as has been demonstrated previously [27]. Additionally, it is possible that some participants with TB but who were asymptomatic in the 2 weeks prior to the home visit were missed, as the symptom screening only collected information about symptoms experienced within the past 2 weeks. Lastly, both at home and the health facilities, sputum production was challenging for some participants with possible TB symptoms, particularly those without cough. Availability of induced sputum services at the referral facilities might have increased our TB detection yield.

The yield of our facility-based TB active case finding may have been impacted by the preceding home-based TB case finding interventions that occurred in each PBIDS site. We would expect that after an intensive community TB screening activity, there would be fewer undiagnosed TB cases seen at the health facilities. However, in both study sites, the yield of the facility-based TB case finding was greater than that of the home-based case finding in terms of both counts and detection rates. Partly, this may reflect that substantial proportions of the PBIDS populations (52 and 64% in Lwak and Kibera, respectively) were not screened at home during the community TB active case finding activities, leaving a number of potential TB cases in the communities undetected. Nevertheless, it may be that the facility-based intensified TB case finding interventions would have had even greater yield if they had not followed rounds of active home-based TB screening.

We also are unable to ascertain whether our TB active case finding activities resulted in additional TB case notifications beyond what would have occurred without these interventions. As noted above, TB cases identified through our study interventions may eventually have been detected through routine, passive facility-based TB case finding. In this circumstance, one might expect that our interventions would produce a temporary bolus of increased TB case notifications (based on earlier detection), followed potentially by a period of decreased case notifications during the timeframe when the TB cases that we diagnosed would otherwise have been detected through passive means. TB case notifications also might have declined in the time period following the study interventions because of reduced TB transmission associated with earlier treatment of infectious TB cases identified during the study than otherwise would have occurred. Alternatively, our intensified TB case finding activities might have resulted in a true overall increase in TB case notifications, if some of the TB cases that we identified would never have been detected otherwise. We do not have information available to determine which scenario has occurred. There are no published TB case notification data that are fully overlapping with the PBIDS populations that we could use to assess changes in notifications over time for our study participants. However, as a future step, we intend to explore the possibility of abstracting data from TB registers at clinics in and around the study sites, and linking those data by name and demographic factors to PBIDS data to identify study participants, to examine trends in TB case notifications in our study populations in the periods preceding, during, and following our intensified TB case finding interventions.

## Conclusion

In summary, we evaluated intensified TB case finding strategies implemented at home and in referral health facilities among persons ≥15 years of age in established rural and urban PBIDS sites in Kenya, as an adjunct to the Ministry of Health’s routine, passive facility-based TB case detection methods. Facility-based intensified TB case finding yielded greater TB detection rates than home-based screening, but both case finding strategies identified high rates of TB among persons living with HIV. Both strategies should be evaluated further for their potential programmatic impact on TB control in appropriate settings.

## Abbreviations

CDC: US Centers for disease control and prevention
CI: Confidence interval
HBCT: Home based counseling and testing
HIV: Human immune-deficiency virus
KEMRI: Kenya medical research institute
MTB: *Mycobacterium tuberculosis*
PBIDS: Population based infectious disease surveillance
PDA: Personal digital assistant
RIF: Rifampicin
RR: Rate ratio
TB: Tuberculosis
